# Tumours of the Breast

**Published:** 1894-12-15

**Authors:** 


### TUMOURS OF THE BREAST.

The question as to the existence of a pre-cancerous stage of epithelial change has lately been discussed by Mr. Raymond Johnson[1] in his lectures at the College of Surgeons on diseases of the breast, and in a paper read by Mr. Cecil F. Beadles at the Pathological Society.[2] Mr. Beadles maintained, as he had done in a previous communication, that in a breast containing a localised carcinomatous growth there were general changes in the epithelium, which he considered were a very early stage of cancerous growth; and he thought recurrent growths often started in the gland tissue left behind after excision of the breast. Mr. Bowlby admitted that there might be inflammatory changes in the breast predisposing to cancerous development, but did not consider the epithelial changes described by Mr. Beadles as an early stage of cancer. Such conditions he had found in non-cancerous breasts even in the involution following lactation. Mr. Raymond Johnson has found this epithelial proliferation in many breasts containing a scirrhus growth, but he has found it also in other breasts, and does not consider it cancerous, though he believes cancer may be more prone to develope in such a gland. It has been suggested that this change in the breast epithelium is secondary to the presence of the growth, but Johnson considers that it is far too general in the gland to admit of such an origin. Heidenhain and Waldeyer are strongly inclined to believe that the growth of a carcinoma in the breast is only a localised change in the epithelium of the same nature as a general epithelial proliferation that is going on all through the gland. They hold, in fact, the view which Mr. Beadles tried to establish. Mr. Stiles, of Edinburgh, and Mr. Watson Cheyne consider that there is no such general and early cancerous change in the epithelium of the breast, but that the disease spreads by the lymphatics only. But there is no doubt the disease may be very widely disseminated by the lymphatics in the breast, and there is always an area of microscopic lymphatic infiltration, around the growth of which there is no other evidence. Hence, although there may be no general cancerous change in the epithelium of the ducts, yet the widespread lymphatic infiltration urgently demands removal of the whole gland in all cases of cancer, however localised. A strong argument against a general cancerous epithelial change seems to us to be the fact that the growth is not usually multiple.

Another question which has engaged attention during the last few months has been the possibility of the transference of an adenoma of the breast into a cancerous growth. Sir James Paget many years ago wrote that he did not consider such a change occurred. Mr. Raymond Johnson, in the lectures already referred to, maintains that no such transference has been proved, and that such is very rare in growths elsewhere, even of a papillomatous nature, which are the ones generally admitted to be liable to such a malignant development. Of course fibro-adenoma is so common in the breast that it would naturally not very rarely be found with cancer in the same breast; and may even be in contact in some cases, as the one recorded by J. Hutchinson, jun., in the transactions of the Pathological Society. But as Johnson points out, we must find the fibro-adenoma actually bursting through its capsule and infiltrating the surrounding breast tissue with microscopic change of tissue before we can fully accept such a transformation. Mr. Warrington Haward, however, records a case[3] in which he believes such a transformation to have occurred. The adenoma was under observation for nine years, during which time it remained stationary, and then rapidly developed a carcinomatous growth. After excision of the breast, a firm fibro-adenoma was found in the upper part, with some small cysts in it. In the skin, where adherent to the tumour, was a button-like nodule of hard carcinomatous growth invading the adjacent part of the tumour, but not penetrating deeply into it. This on histological examination proved to be scirrhus, and the fibro-adenoma showed only the usual structure of such growths. This subcutaneous scirrhus growth had been present for six months before removal.

Much has been written about the nature of duct cancer of the breast during the last few years, and many cases reported at meetings of the Pathological Society. Mr. Bowlby has recorded several,[4] and the general characters of the growth are now fairly well known. They are essentially of a papillomatous nature, and although they do sometimes recur locally, are not otherwise malignant, unless their tendency to infiltrate the fatty tissue around is considered sufficient proof of malignancy. Many grow for many years before removal. A case of the kind referred to is recorded by Mr. Charles A. Morton in the *Bristol Medico-Chirurgical Journal*.[5] The growth was very large, and recurred locally two years after its first removal. After removal the tumour measured five and a half by four inches. On cutting into it, dark red blood poured out in large quantity. It consisted of a number of cysts containing either a very soft white papillomatous growth or altered blood-clot. The papillomatous growth was as soft as velvet, and easily washed away from the interior of the cyst by a gentle stream of water. Microscopic section showed the typical structure of papilloma. Although the tumour was so large it had not infiltrated the surrounding tissues or infected the glands. Mr. Morton does not consider that the characters of these tumours are sufficiently malignant to justify the name "duct cancer."

Another case of the same kind is reported[6] from the Melbourne Hospital by Dr. Moore. In this case also the growth was one of considerable size. It was of five years' duration, in a patient seventy years of age.

[1] Lancet, June 9th, 1894. [2] Brit. Med. Journ., Vol i., 1894, p. 635. [3] Lancet, 1894, Vol. i., p. 1,245. [4] Lancet, June 10th, 1893. [5] March, 1894. [6] Lancet, Vol. i., 1894, p. 1,448.

